# Evaluation of Pseudo-Haptic Interactions with Soft Objects in Virtual Environments

**DOI:** 10.1371/journal.pone.0157681

**Published:** 2016-06-28

**Authors:** Min Li, Sina Sareh, Guanghua Xu, Maisarah Binti Ridzuan, Shan Luo, Jun Xie, Helge Wurdemann, Kaspar Althoefer

**Affiliations:** 1 School of Mechanical Engineering, Xi'an Jiaotong University, Xi'an, Shaanxi, China; 2 State Key Laboratory for Manufacturing Systems Engineering, Xi'an Jiaotong University, Xi'an, Shaanxi, China; 3 Department of Aeronautics, Imperial College London, London, United Kingdom; 4 Centre for Robotics Research, Department of Informatics, Kings College London, London, United Kingdom; Shanghai Jiao Tong University, CHINA

## Abstract

This paper proposes a pseudo-haptic feedback method conveying simulated soft surface stiffness information through a visual interface. The method exploits a combination of two feedback techniques, namely visual feedback of soft surface deformation and control of the indenter avatar speed, to convey stiffness information of a simulated surface of a soft object in virtual environments. The proposed method was effective in distinguishing different sizes of virtual hard nodules integrated into the simulated soft bodies. To further improve the interactive experience, the approach was extended creating a multi-point pseudo-haptic feedback system. A comparison with regards to (a) nodule detection sensitivity and (b) elapsed time as performance indicators in hard nodule detection experiments to a tablet computer incorporating vibration feedback was conducted. The multi-point pseudo-haptic interaction is shown to be more time-efficient than the single-point pseudo-haptic interaction. It is noted that multi-point pseudo-haptic feedback performs similarly well when compared to a vibration-based feedback method based on both performance measures elapsed time and nodule detection sensitivity. This proves that the proposed method can be used to convey detailed haptic information for virtual environmental tasks, even subtle ones, using either a computer mouse or a pressure sensitive device as an input device. This pseudo-haptic feedback method provides an opportunity for low-cost simulation of objects with soft surfaces and hard inclusions, as, for example, occurring in ever more realistic video games with increasing emphasis on interaction with the physical environment and minimally invasive surgery in the form of soft tissue organs with embedded cancer nodules. Hence, the method can be used in many low-budget applications where haptic sensation is required, such as surgeon training or video games, either using desktop computers or portable devices, showing reasonably high fidelity in conveying stiffness perception to the user.

## Introduction

Haptic feedback aims to create a sensation of touch to the user when interacting with a remote or virtual object. It enables users to manipulate objects and perform complex tasks in virtual environments in a more realistic sense and with higher accuracy. A number of haptic devices have been widely used in research, including Geomagic device series (3DS Inc.) [1], Delta, Omega and Sigma haptic systems (Force Dimension Inc.) [2], the haptic system from Novint Technologies, Inc. [3], the Haptic Interface Robot (HIRO) device [4], and Rutgers Master II force feedback glove [5]. One of the disadvantages of using haptic devices to provide force feedback is that haptic devices are relatively costly [6]. There is an observable trend in creating low-cost haptic gaming devices and training systems for medical students [7–9]. Low-cost haptic feedback on consumer devices such as tablet computers and smart phones has become a solution. More and more consumer devices acquire user contact force information from the screen surface exploiting an integrated pressure-sensitive technology while force feedback is currently missing.

Vibration, as a low-cost substitute of force feedback, is commonly used to simulate haptic or tactile feelings of textures for interactive devices and are proven to be effective means for conveying haptic information [10–13]. Vibration feedback is currently available in off-the-shelf consumer devices such as tablet computers and smart phones.

**Fig 1:**
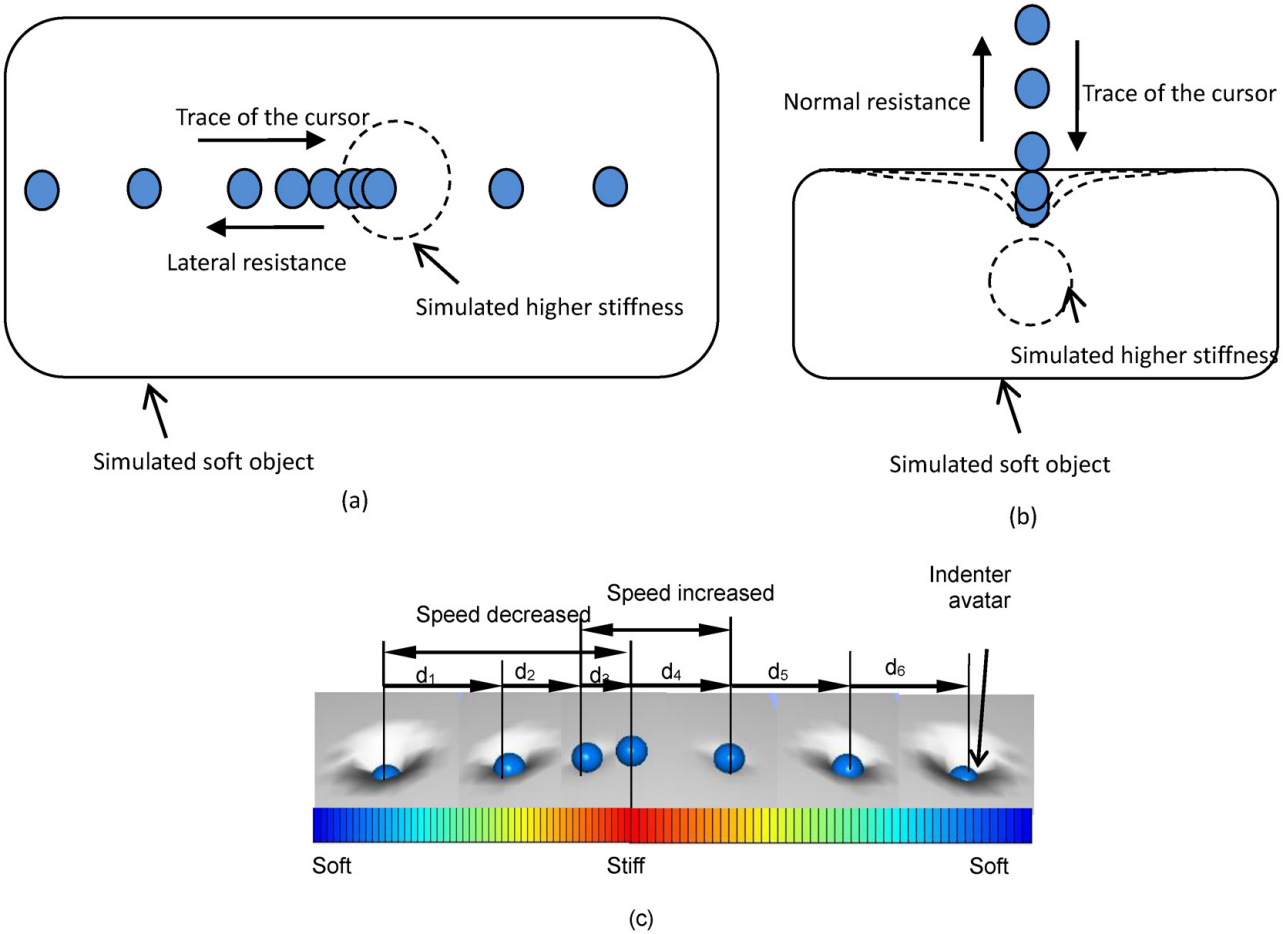
Pseudo-haptic feedback for soft surface stiffness simulation. (a) Simulated lateral resistance when the indenter approaches an area with higher stiffness; (b) simulated normal resistance when the indenter presses on the soft surface area with higher stiffness; (c) modification of the surface deformation and indenter avatar speed when passing over an area with higher stiffness (d1>d2>d3, d3<d4).

Pseudo-haptic feedback is another low-cost haptic feedback solution for consumer devices creating an illusion of force and haptic feedback using only visual information [14–22]. An interaction between a human finger and a soft object can deform the surface of the object. The change in the indentation depth and the deformation of the surface provides clues on the stiffness property of the surface. (Note that stiffness is a subjective impression of the physical deformability and compressibility of objects). In our previous research, we proposed to simulate the stiffness of a soft surface using a desktop computer with a computer mouse [23], tablet computers [24], or touchpads [24] employing the principle of pseudo-haptic feedback. The method exploits a combination of two feedback techniques: soft surface deformation visualization and control of the indenter avatar speed (see Fig 1). As the indenter approaches an embedded hard nodule in soft object during palpation a lateral resistance to motion was simulated by reducing the ratio between the indenter avatar (cursor) displacement and the input device (computer mouse) displacement, as demonstrated in Fig 1(a). The effectiveness of our method in virtual hard nodule detection has been preliminarily proved [23,24]. However, the effect of these two feedback techniques in stiffness presentation has not been examined individually. Moreover, the ability of our method in distinguishing different sizes of virtual hard nodules integrated into the simulated soft bodies has not been investigated.

Multi-fingered interactions are more common than single-fingered interactions in daily life when attempting to explore the properties of surfaces of soft objects. Multi-fingered haptic feedback using actuators has been proved to be more efficient than single-fingered one in conveying haptic information as described in our previous research [25,26]. Pseudo-haptic feedback can simulate similar effect using multiple indenter avatars, and hence, a wider surface area can be investigated during one indentation. Moreover, the user can conveniently compare the stiffness values at different locations by observing the differences of indentation depths of the separate indenter avatars. Thus, the interactive experience can be further improved.

In order to provide guidelines for the further development of stiffness simulation of soft surfaces, in this paper we compare two pseudo-haptic feedback techniques: visual feedback of soft object deformation and sliding resistance displayed by appropriately slowing down or accelerating the cursor speed. The performance of human participants in distinguishing different sizes of virtual hard nodules integrated into the simulated soft bodies is evaluated confirming the effectiveness of our pseudo-haptic feedback method. We evaluate the advantages of multi-point pseudo-haptic feedback over single-point pseudo-haptic feedback. In order to prove our proposed method to be an effective alternative to vibration feedback in current off-the-shelf consumer devices, the pseudo-haptic feedback system is benchmarked against vibration feedback provided by a tablet computer.

This paper is organized as follows. The second section explains the concept and algorithm of interactive haptic display and single-point pseudo-haptic feedback for soft surface stiffness simulation, constitutes our multi-point pseudo-haptic feedback method, and describes the vibration feedback method used in the comparison study. User study section describes the validation test protocol. The fourth section presents the results of our studies. Conclusions are drawn in the last section.

## Haptic Feedback Methods

### Single-point pseudo-haptic interaction

Our pseudo-haptic-based feedback approach utilizes an indenter avatar speed control strategy, to evoke the perception of tangential resistance (due to increased local stiffness) to sliding finger motion across an object’s surface. The trace of the indenter avatar movement over equal time intervals is shown in Fig 1(c). When the indenter avatar slides over a soft object area in which the stiffness value is higher than the surrounding areas, our pseudo-haptic feedback system displays surface deformations with a reduced indentation depth, i.e., the user gets the impression that his or her finger is “pushed” upwards by the locally increased stiffness.

This paper involves two types of pseudo-haptic systems. The first one consists of a virtual model of a soft object, an indenter avatar displayed on a computer monitor, and a computer mouse used as an input device. The second one is formed from a pressure-sensitive tablet computer, a virtual model of a soft object, an indenter avatar displayed on the tablet screen, and an S-pen used as an input device.

**Fig 2:**
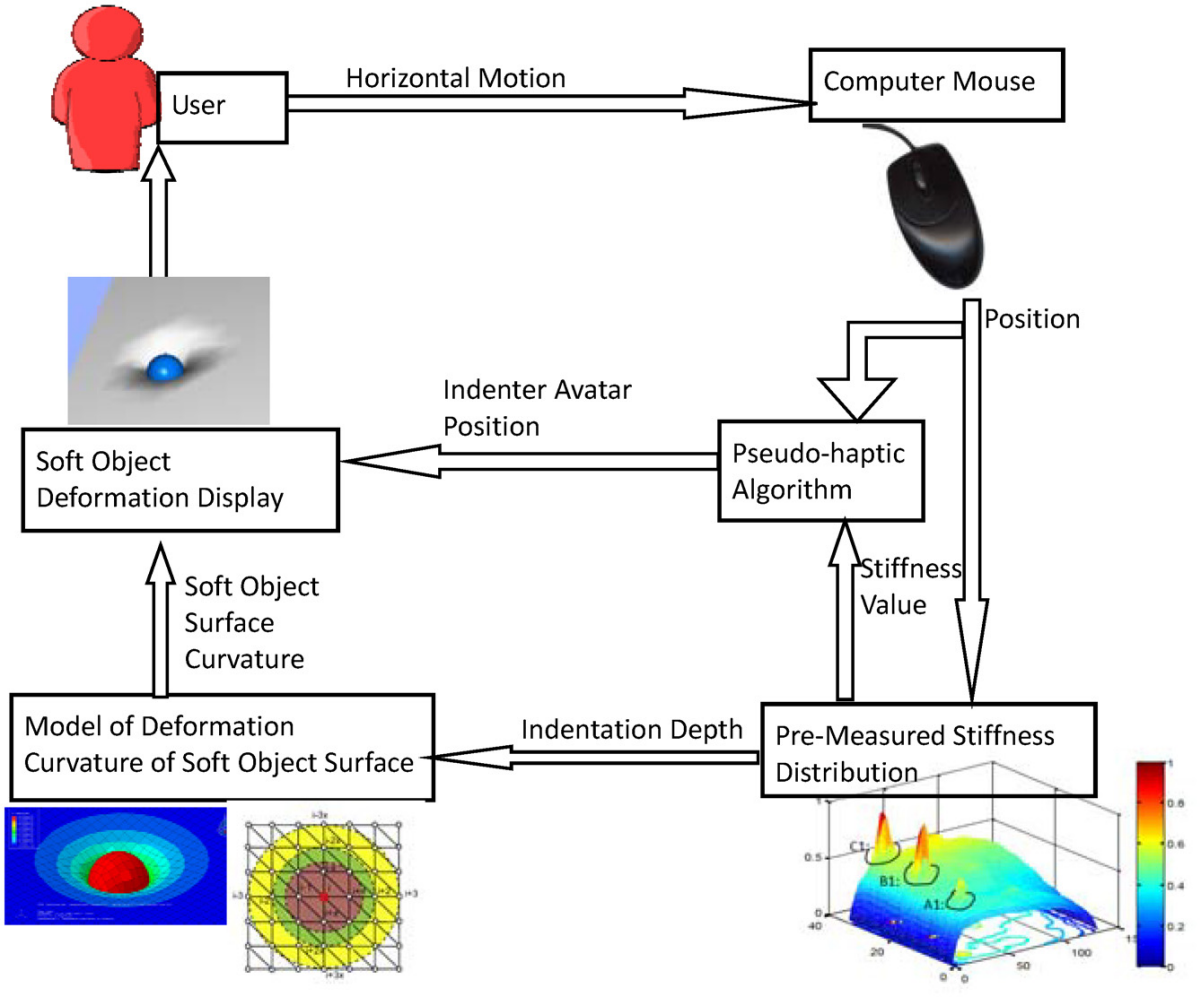
Schematic diagram of the pseudo-haptic surface stiffness simulation using a desktop computer.

For the first case, the applied force is assumed to be the same during the entire interaction process. Local stiffness values are computed according to the position information of the indenter avatar and a predefined stiffness distribution (see Fig 2). The indentation depth is calculated based on the stiffness value. Then, the soft surface deformation is obtained by modifying the nodes’ heights according to the indentation depth and the geometry of the used deformable soft surface model. The details of the real-time deformable model for hyperelastic materials are presented in [27].

If the object’s surface is displayed in the application window as a plane parallel to the tablet surface, any surface deformation (which would be then perpendicular to the tablet surface) is difficult to be appreciated by the user. In Kobubun et al. [21], a black and white polygons-formed surface was used to visualize surface deformation. In order to convey three-dimensional spatial information, here, the surface is displayed using a perspective view with an angle of 45° towards the user and deformations are emphasized using shadow effects. The original computer mouse pointer is hidden when the computer mouse moves inside the application window. The coordinates of the application window are linearly mapped to the soft surface. A blue sphere as an indenter avatar is then displayed at the corresponding position on the soft object.

As shown in Fig 2, a predefined 2D stiffness distribution (see more details in [23,28,29]) is assigned to the soft object surface. Thus, stiffness level values are mapped to the corresponding surface coordinates. Cursor speed *CursorSpeed* is calculated according to the following equetions

$$CursorSpeed_{n}=\{\begin{array}{c} CursorSpeed_{n-1}\\ CursorSpeed_{n-1}\times (1.1-S_{n})/(1+|Ds|\\ CursorSpeed_{n-1}/(1.1-|Ds| \end{array})\begin{array}{c} \left(S_{n}=S_{n-1}\right)\\ \left(S_{n}>S_{n-1}\right)\\ \left(S_{n}<S_{n-1}\right) \end{array}\;,$$ (1)

Where *S*_n_ is the stiffness level at current cursor position; *S*_n-1_ is the stiffness level at previous cursor position; *Ds* is the stiffness level difference between current and previous cursor positions; *CursorSpeed*_n-1_ is cursor speed at previous step.

**Fig 3:**
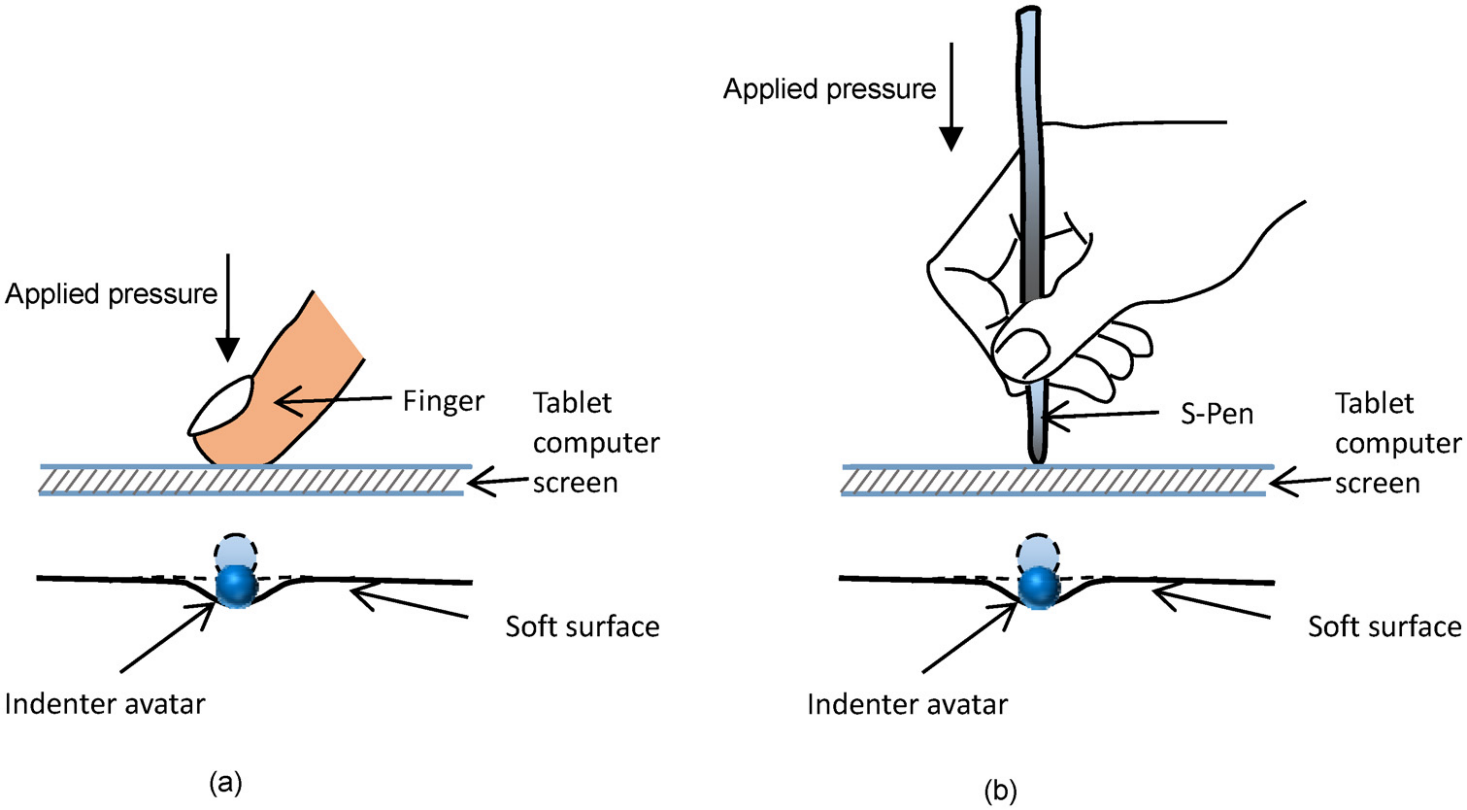
Concept of interactive haptic display using tablet computers.

Using pressure-sensitive tablet computers, not only can the two-dimensional motion input be captured but also the normal indentation force. Therefore, in this case, the applied pressure is not assumed to be constant any more. The user touches the tablet computer with a special force-sensitive pen (S-pen) or a bare finger providing two-dimensional movement kinematics and a normal force (see Fig 3). Initially, the indenter avatar is positioned just above an arbitrary location on the soft surface. As the user touches the screen, the indenter avatar follows the point of interaction between user finger and chosen screen location. When the user adds pressure on the screen, the indenter avatar moves downwards and causes the virtual soft object and its surface to deform. As the pressure increases, the deformation increases. Therefore, the inputs and outputs of the system can be summarised as follows:

- Inputs:
  1. The estimated stiffness from the model given the tip position.
  2. Two-dimensional movement kinematics on the tablet screen.
  3. Applied normal pressure on the tablet screen.
- Outputs:
  1. The normal reaction force from the tablet computer.
  2. The virtual resistance along the movement direction.
  3. The soft surface deformation shown on the graphical interface.

The indentation depth is controlled according to a combination of the applied touch pressure and the surface stiffness at the interaction point. The deformation of the soft surface is calculated based on the indentation depth, the model of soft object’s deformation curvature [27], and current avatar location. Unlike using a desktop computer with a computer mouse, both the haptic (contact reaction force from the device surface) and visual (the indenter avatar speed and surface deformation) cues are presented at the same active point of interaction. Therefore, tablet computer may provide a more natural interaction experience than desktop computer with a computer mouse.

In our previous study, we demonstrated that by using a Samsung Galaxy Note 10.1 and an S-pen, which provides indentation and 2D motion input, we have a better performance in nodule detection as compared to when using a Motorola Xoom which is operated with the bare finger [24]. Therefore, in this study, we only use the better performing device, the Samsung Galaxy Note 10.1 with its S-pen for input. Samsung Galaxy Note 10.1 is a quad-core tablet with 1024 levels of pressure sensitivity with a 10.1 inch HD Display and an Android operating system (here: v4.1.1 (Jelly Bean)). The overall dimensions of the tablet are 262×180×8.9 mm^3^. The force level-force value relationship of the pressure sensitive screen is described as

$$f_{n}=0.1008e^{4.2081f_{l}}\;,$$ (2)

where *f*_l_ is the force level read by using getPressure () method in Android SDK and *f*_n_ is the corresponding normal force [24].

The indenter avatar speed is varied by adding a time delay to the rendering task of the indenter avatar when it is approaching a stiffer area. When the indenter passes over the stiffer area, the indenter avatar continues to follow the contact point. The delay time is expressed as

$$t_{d}=\Delta f_{t}\cdot m\;,$$ (3)

where *f*_*t*_ is the value of the tangent reaction force, *f*_*tn-1*_ is the tangent reaction force value at previous avatar position, *Δf*_*t*_ is the reaction force difference (Δ*f*_*t*_ = *f*_*tn*_ − *f*_*tn-*1_), and *m* is a scalar value. The values of the reaction force matrices were obtained during a previous experiment where a rolling indenter was rolled over a silicone block [23,30]. The minimum delay time should be set higher than the time interval between frame updates so that the user can notice the indenter avatar when it lags behind the contact point. The frame interval for this program is 30 ms. An afterimage is thought to persist for approximately 40 ms on the retina due to the phenomenon of persistence of vision of the eye. Therefore, the delay time needs to be set to be longer than 40 ms. In this study we set to be 50 ms. Moreover, it is designed that the user should notice the minimum reaction force difference of 0.1 N in in this system. Thus, m is 50/0.1 = 500. The calculated delay time was then added to the program frame interval time.

### Multi-point pseudo-haptic interaction

**Fig 4:**
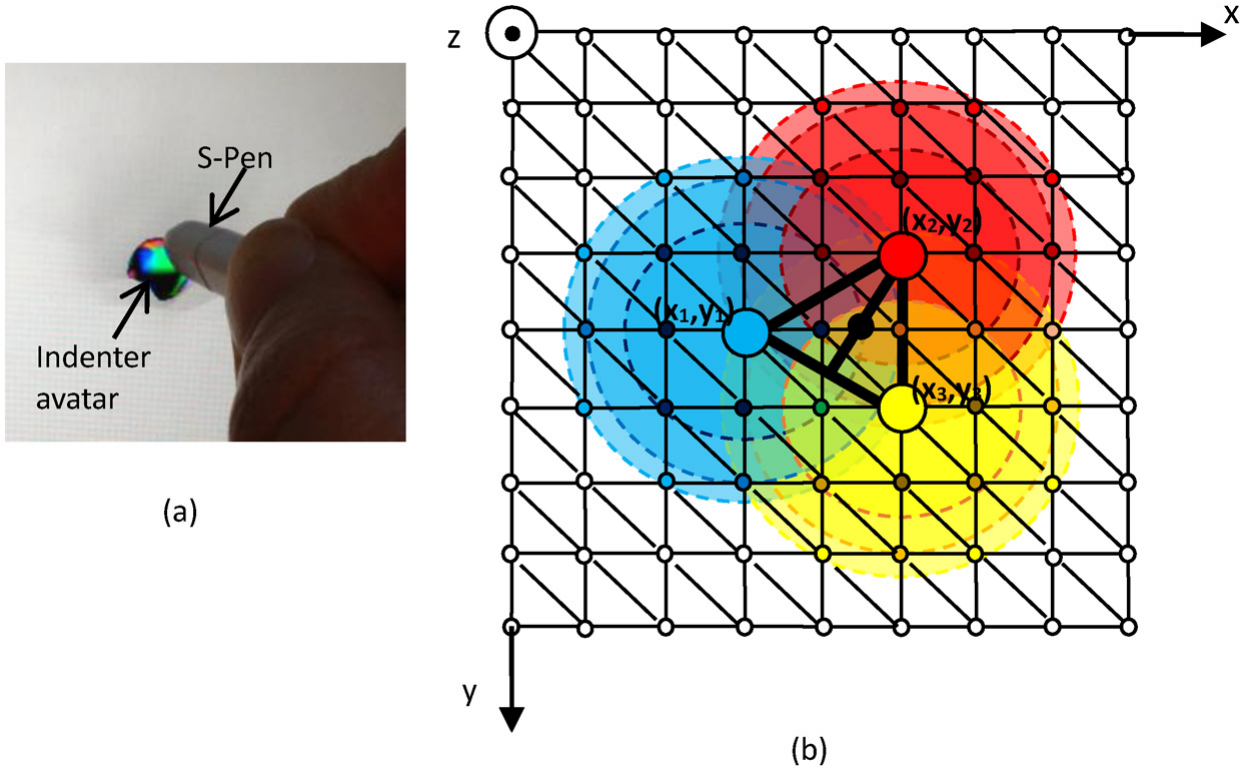
Single-point and multi-point soft surface stiffness simulation. (a) A single indenter avatar represents one interaction point; (b) the locations of the three indenter avatars (marked using blue, red, and yellow dots), barycenter of the triangle (marked using a black dot), and the affected soft surface nodes of each indenter avatar (marked using small blue, red, and yellow circles).

To simulate multi-fingered interaction with soft object surfaces, three indenter avatars are aligned in a triangular fashion during the operation, as shown in Fig 4(b). Horizontal relative positions (x and y directions) of these three indenter avatars are fixed. The barycenter of the triangle (marked using a black dot in Fig 4(b)) is set to follow the user’s motion and the perpendicular input force (along z-direction) applied by the user is divided equally to the three indenters. Thus, these three indenters move uniformly in the x and y directions but translate independently from each other in the z-direction according to the stiffness value of the object under the surface. This approach enables the user to explore and examine three neighboring soft surface areas simultaneously using three fingers. In the soft surface deformation modeling method [22], indentation depth is divided into four ranges for four different cases, where the demarcation points are (2-√3) · *r*/2, *r*, and 2*r*. The influenced node neighborhoods are defined according to the indentation depth. The colored circles in Fig 4(b) show the affected nodes of the four cases. Note that when the indentation depth is less than (2-√3) · *r*/2, only the node at the center of indentation is adjusted. The number of the affected vertices of the triangle increases as the indentation depth increases. In the neighborhood of each indenter avatar, the height values of the deformable object’s nodes are adjusted according to the z-direction input of the indenter avatar (i.e., the pressure provided by the user) and the lateral distance between these nodes and the indenter avatar. Note that the average value of height is applied when the node of the deformable soft surface model is located in the influenced neighborhoods of more than one indenter avatars, but may differ owing to the calculated height values that are a function of the stiffness values at these indenter avatars’ locations (see Fig 4(b)). If the stiffness values at these indenter avatars’ locations are the same, these indenter avatars are at the same height, namely *z*_1_ = *z*_2_ = *z*_3_. When the deformations at the three points are different, one can easily comprehend the variation in stiffness values from height differences of these indenter avatars (*z*_1_, *z*_2_, *z*_3_). In order to prevent the S-pen or the finger of the user from obstructing the view, the indenter avatars are displayed at a distance of 15 mm from the interactive point.

### Vibration feedback for haptic interaction

The Samsung Note 10.1 has a vibration motor (model number GH59-11990A) located behind its touch screen. Vibration feedback is added when the S-pen is approaching an area with a hard nodule underneath, i.e. when the user moves from a softer to a stiffer region. Our pseudo-feedback approach is intended to make users “feel” as if they have hit something hard beneath the surface. The vibration time duration is adjusted proportionally to the delay time of the avatar’s lateral motion as part of single-point pseudo-haptic feedback (as described in Section 2.1, the indenter avatar speed is varied by adding a time delay to the rendering task of the indenter avatar when it is approaching a stiffer area).

## User Study

### Experimental protocol

We examined how human participants interact with virtual soft objects using different feedback modalities. Through this user study, the participants were able to explore the surfaces of soft objects and observe the changes in surface deformation and/or the movement speed of the indenter avatar(s) to determine if there was a hard inclusion underneath the surface. The purposes of the experimental validation study include: (a) compare two pseudo-haptic feedback techniques: visualization of soft surface deformation and modification of indenter avatar speed; (b) evaluate the efficiency of the proposed single-point pseudo-haptic feedback in hard nodule size discrimination; (c) verify the advantages of using a multi-point pseudo-haptic feedback approach when compared to a single-point haptic feedback mechanisms; and (d) compare our pseudo-haptic feedback approach and currently available vibration-based haptic feedback in tablet computers. This work was approved by the King’s College London Biomedical Sciences, Dentistry, Medicine and Natural & Mathematical Sciences Research Ethics Subcommittee (BDM/10/11-95). Participants were required to have normal or corrected vision and intact haptic sensing abilities. All participants provided signed written informed consent to participate in this study.

User experience with interactive virtual environments can be characterized according to the required time for completion of the interactive task for the participant. Therefore, during the user-experience test a stopwatch was used to measure the time required by each participant to explore the surface and come to an answer in each trial. The instrument allowed a precision of the time measurement of ±1 s. All the tests were performed sequentially and pseudo-randomly by each participant (each participant starts at a different test sequentially). A test would start once the participant understood and felt comfortable with the procedure of the experiment.

#### Single-point interaction study

**Fig 5:**
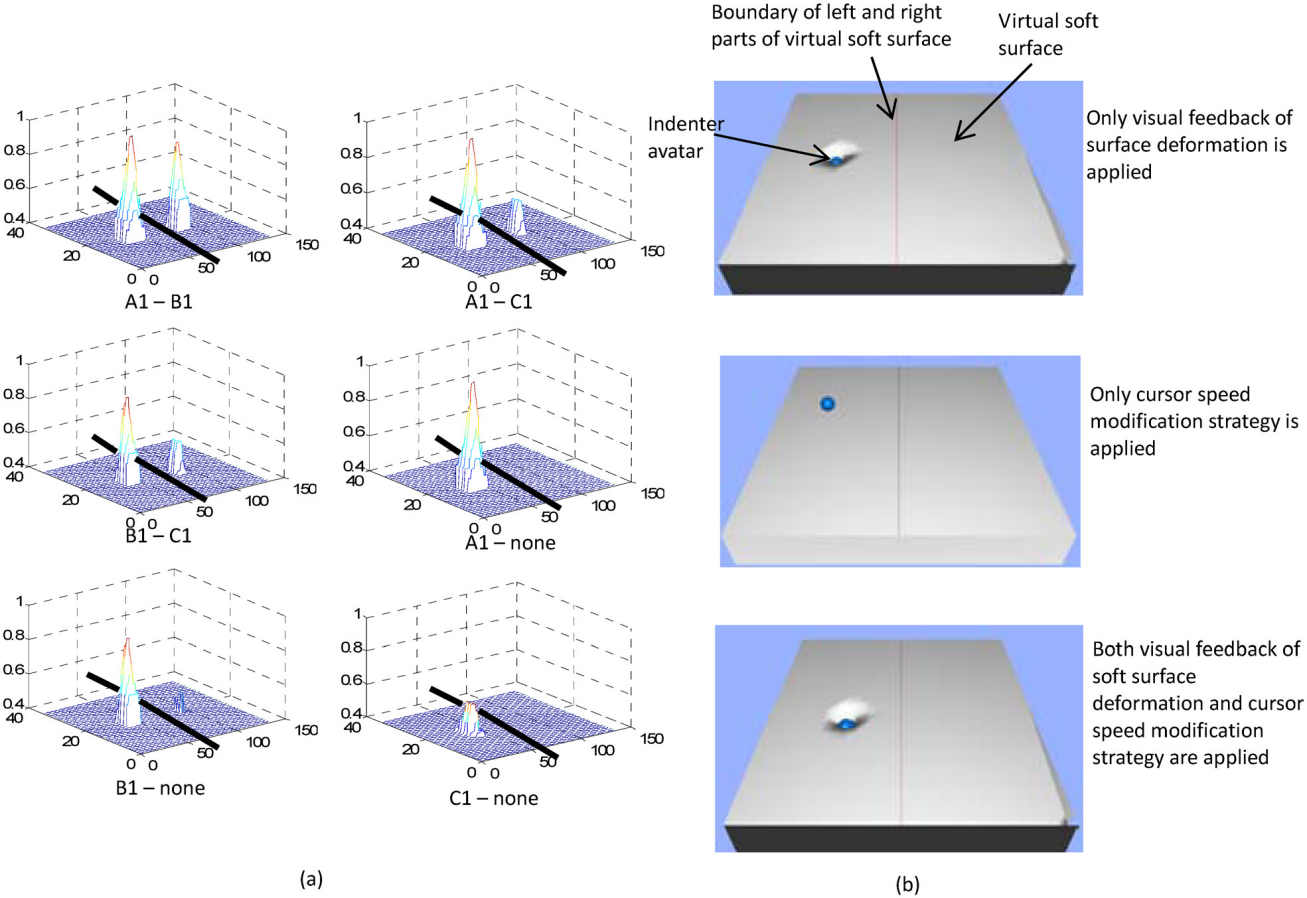
Comparison experiment of pseudo-haptic feedback techniques. (a) The stiffness distribution information used in our experiment; the surface is divided into left and right parts; four types of status (A1, B1, C1 and no hard inclusion buried inside) are possible at each side; thirteen status combinations at the two sides are considered, and (b) the user interfaces of the three feedback modalities.

As shown in Fig 5, three pseudo-haptic feedback techniques including visual feedback of surface deformation of such virtual soft objects, modification of cursor speed, and the combination of the two were examined. In this experiment, a red line divided the virtual surface into two parts: left and right (see Fig 5(a)). In each part, there were two status possibilities for hard inclusion buried inside: a hard inclusion exists (A1, B1, or C1, A1>B1>C1) or no hard inclusion exists (‘none’ for short in the following text). Thirteen sets of stiffness distribution data were used in this experimental study. During the user study, participants were asked to explore the virtual surface by using a computer mouse. Fourteen participants aged from 21 to 36 were involved in the trials: one woman and thirteen men. At last, participants were asked to choose their favorite pseudo-haptic feedback technique from visual feedback of surface deformation, modification of cursor speed, and the combination of the two.

In the experiment of nodule size discrimination, the stiffness distribution matrices used in the soft surface stiffness simulation came from experiments with a 120×120×25 mm^3^ silicone block with three spherical nodules (A, B, and C, A>B>C) buried inside [24]. In this experiment, twenty participants (6 females, 14 males) aged from 20 to 30 participated. During the user study, participants were asked to explore a virtual surface by using a tablet computer with an S-pen. Buried nodules were marked on the surface using blue dots. Participants were asked to compare the stiffness of the three locations (A, B, and C) and sort the stiffness levels (A>B>C). The stiffness level order of the three hard nodules and the elapsed time were then recorded. When participants successfully sorted every two nodules, they were scored one point.

#### Single-point interaction vs. three-point interaction and vibration

The same participants as nodule size discrimination experiment involved in this experiment. Participants were asked to explore the surface and to mark the hard nodules buried inside (A, B, and C) with blue dots. When participants successfully localized a nodule, they were scored one point. Three feedback modalities were provided: 1) single-point pseudo-haptic feedback, 2) multi-point pseudo-haptic feedback, and 3) vibration feedback. It is worth mentioning that the same stiffness distribution (i.e., spatial distribution of nodules within a silicon phantom) was used throughout nodule size discrimination and nodule localization experiments. However, the orientation of the stiffness map was different from test to test, thus the participants would not learn the nodules’ locations from the earlier tests.

### Data processing methods

Sensitivity *Se* [31], which indicates the test's ability to identify positive results, specificity *Sp* [31], which indicates the test’s ability to identify negative results, and accuracy *ACC* [32] were used as measures of the nodule detection performance of the palpation methods. Wilson score intervals [33], which have good properties even for a small number of trials (less than 30) and/or an extreme probability, were calculated for *Se*, *Sp*, and *ACC* at a 95% confidence level. Shapiro-Wilk test [34] was used to check the sample normality. When the sample normality was confirmed, student t-test with Bonferroni correction was applied for pairwise comparisons. Otherwise, Kruskal-Wallis rank sum test [35] was used to check stochastic dominance among test groups and Dunn’s test [36] with Bonferroni adjustment was used for multiple pairwise comparisons. The significance level was set equal to 0.05.

## Results and Discussion

### Comparison of deformation visualization and cursor speed modification

**Fig 6:**
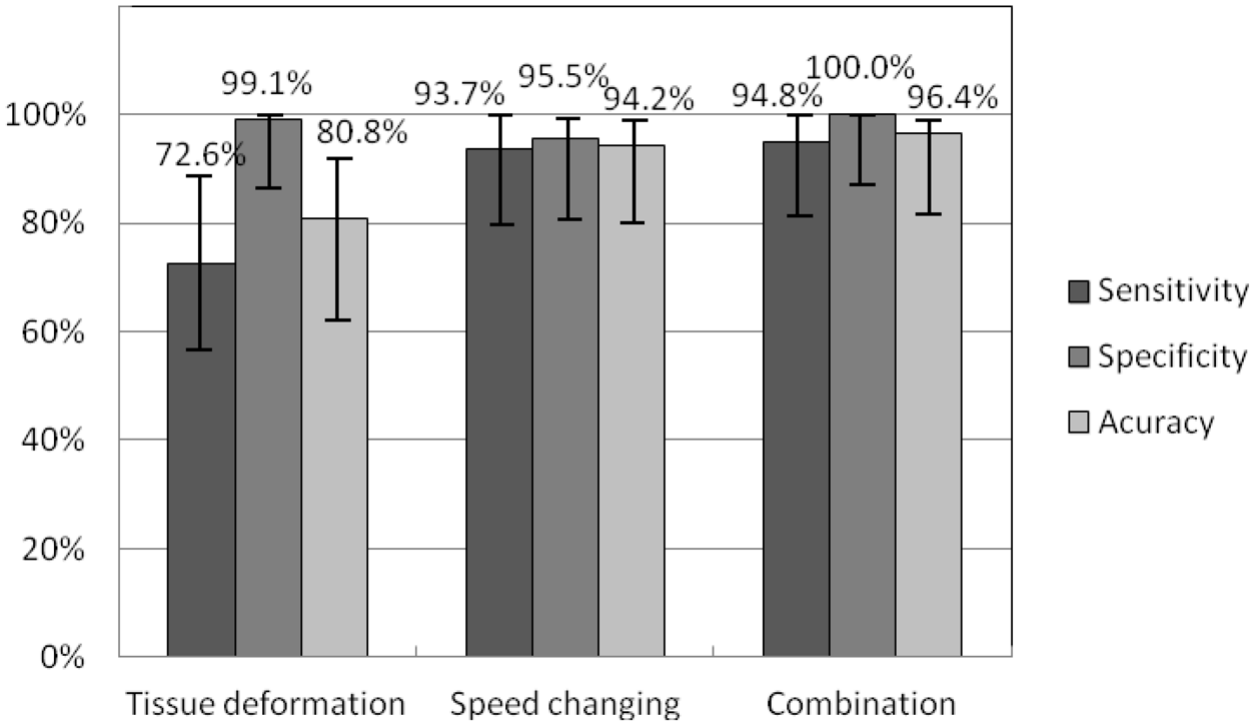
The nodule detection sensitivity, specificity and accuracies with Wilson score intervals at a 95% confidence level of visual feedback of surface deformation, speed modification strategy, and combination of the two feedbacks.

Fig 6 presents the nodule detection sensitivities *Se*, specificities *Sp*, and accuracy *ACC* obtained by using different pseudo-haptic interaction techniques. The sample size was 28 (2 nodules × 14 participants). As shown in Fig 6, the combination of modification of the cursor speed and the visual feedback of surface deformation had the highest nodule detection results for parameters *Se*, *Sp* and *ACC*, namely 94.8% (95% confidence interval: 80.0%– 98.9%), 100% (95% confidence interval: 87.9%– 100%), and 96.4% (95% confidence interval: 82.3%– 99.4%), respectively. Pseudo-haptic feedback using the speed modification strategy resulted in higher values of *Se* (93.7% vs. 72.6%) and *ACC* (94.2% vs. 80.8%) than the visual feedback of surface deformation. However, the situation was reversed regarding *Sp* (95.5% vs. 99.1%).

Kruskal-Wallis chi-squared test results of *Se* (*Х*^2^ = 18.482, *p* = 9.699×10^−5^) and *ACC* (*Х*^2^ = 16.895, *p* = 2.144×10^−4^) showed that at least one sample stochastically dominates another sample. Dunn’s test with Bonferroni adjustment was then performed and the test result is shown in Table 1. It can be seen that the *Se* and *ACC* of the tests using the speed modification strategy and the combination of the speed modification strategy and visual feedback of surface deformation were significantly higher than the corresponding results when using visual feedback of surface deformation. Regarding *Sp*, there was no significant difference among the tests (*Х*^2^ = 2.151, *p* = 0.341). After this experiment, ten participants (71.4%) preferred the combined feedback technique over the others; two (14.3%) claimed that the combined feedback technique and the visual surface deformation feedback were the same and better than the speed control feedback technique; one (7.1%) preferred the visual surface deformation feedback; one (7.1%) claimed that the speed control feedback technique was the quickest feedback technique. Overall, the statistical analysis results show that the combined feedback technique performed the best.

**Table 1:** Comparison of sensitivity and accuracy.

	Item	*Z*	*p*
*Se*	Deformation & Speed Control	-3.637	0.0004**
	Deformation & Combination	-3.040	0.0002**
	Speed Control & Combination	-0.167	1.0000
*ACC*	Deformation & Speed Control	-3.031	0.0037**
	Deformation & Combination	-3.920	0.0001**
	Speed Control & Combination	-0.889	0.5612

**. Stronger significance than at the 1% level

**Fig 7:**
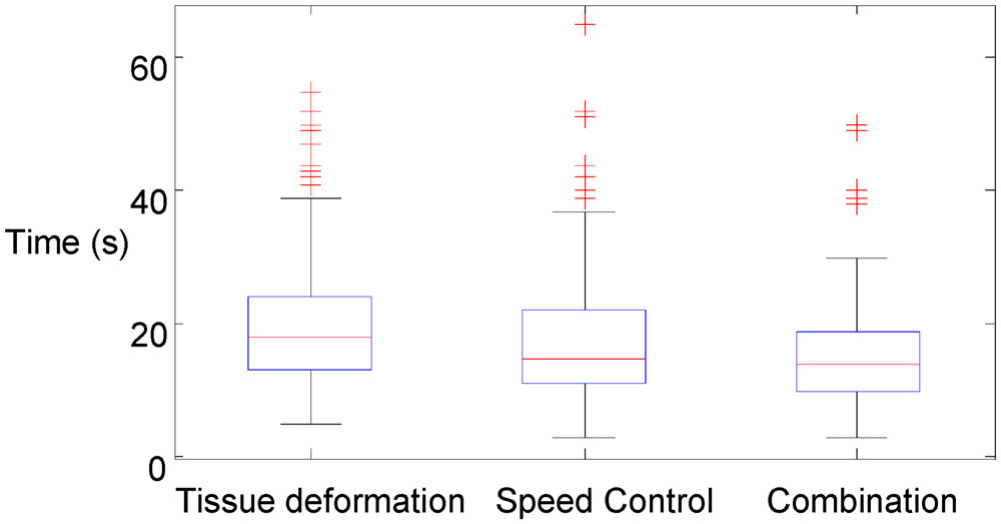
Time used for nodule detection using visual feedback of surface deformation, speed control strategy, and combination of the two feedbacks.

Fig 7 presents the elapsed time during nodule identification tests. The test results of Shapiro-Wilk normality test showed that the elapsed time (Tissue deformation: *W* = 0.885, *p* = 1.374×10^−10^; Speed control: *W* = 0.872, *p* = 2.559×10^−11^; Combination: *W* = 0.876, *p* = 4.069×10^−11^) had a normal distribution. A student t-test with Bonferroni correction was performed to compare the elapsed time during the tests. Table 2 shows the test results. The combined feedback modality required significantly less time to complete the task than the other two feedback modalities.

**Table 2:** Student T-test for elapsed time.

Item	*p*-value
Deformation & Speed Control	5.22×10^−4^**
Deformation & Combination	2.85×10^−8^**
Speed Control & Combination	0.0481*

*. Significant at the 5% level;

**. Stronger significance than at the 5% level

### Nodule size discrimination

The overall discrimination rate was calculated as 81.7% (95% confidence interval: 70.1%-89.4%). When the largest nodule size difference was used (the pair A and C), the highest discrimination rate was calculated as 90% (95% confidence interval: 69.9%-97.2%) where the participants could correctly identify that nodule A was larger than nodule C. Also, 85% (95% confidence interval: 64.0%-94.8%) of the participants could correctly discriminate nodules A and B and 70% (95% confidence interval: 48.1%-85.5%) of the participants could correctly distinguish nodule B and C. The average perceived size order of nodule A, B, and C were 1.25, 2.15, and 2.6, respectively, which showed a good match to the real order of the nodule sizes.

### Nodule localization

**Fig 8:**
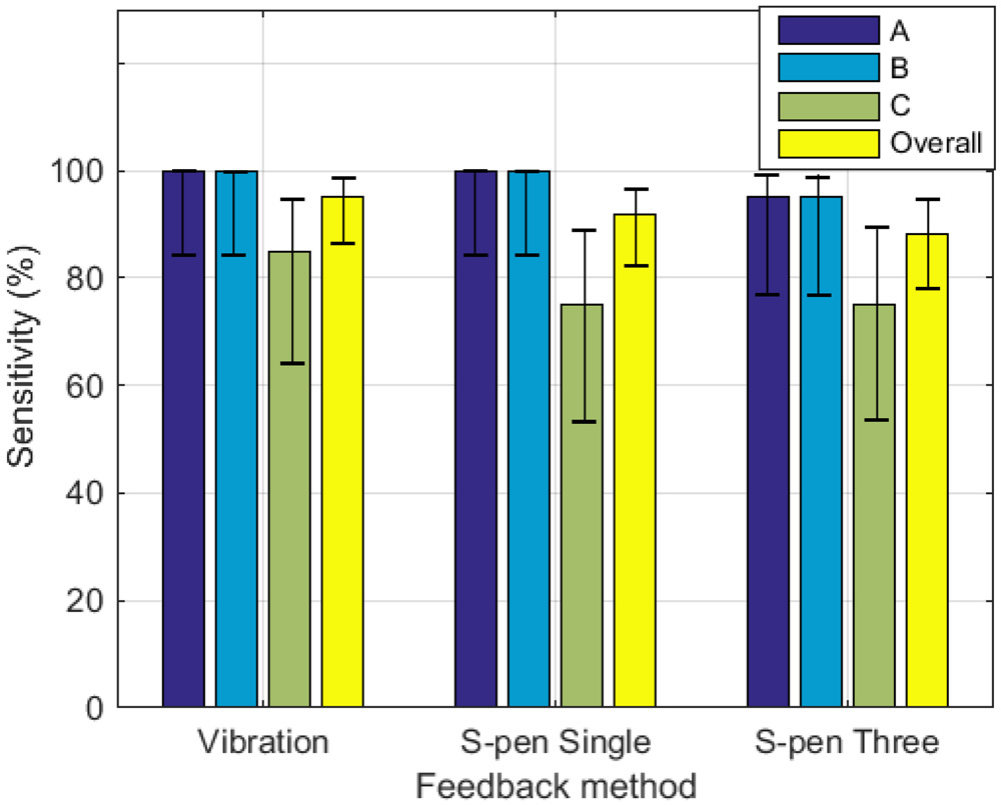
Nodule detection sensitivities with Wilson score intervals at a 95% confidence level of vibration feedback, single-point pseudo-haptic feedback, and multi-point pseudo-haptic feedback.

Fig 8 presents the nodule detection sensitivity *Se*. Single-point pseudo-haptic interaction had a higher *Se* (91.7%, 95% confidence interval: 82.0–96.4%) than the multi-point pseudo-haptic interaction (88.3%, 95% confidence interval: 77.8–94.2%). The sensitivity of vibration feedback was calculated as 95% (Wilson score interval at a 95% confidence level: 91.5%-97.1%). There was no significant difference in the performance of nodule detection *Se* among the tests (*Х*^2^ = 4.352, *p* = 0.226).

**Fig 9:**
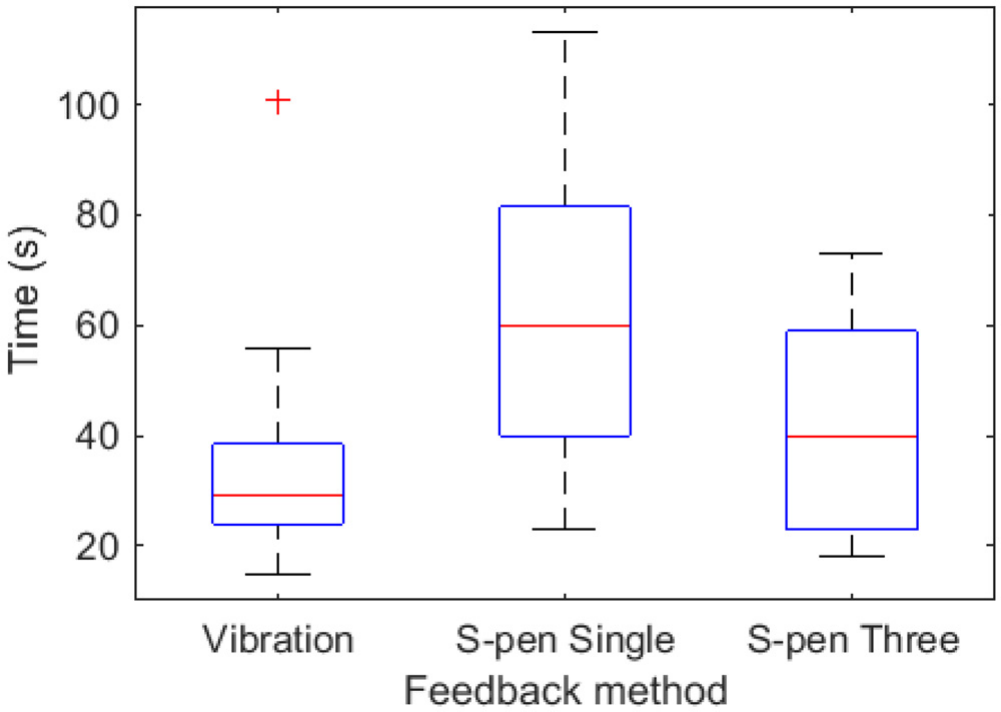
Elapsed time of nodule detection using vibration feedback and pseudo-haptic feedback.

Fig 9 shows the elapsed time for nodule detection for the three feedback modalities. Among the three modalities, the nodule detection approach in an interactive virtual environment with vibration feedback required the shortest time (33.95s, SD = 18.97). The elapsed time was (41.65 s, SD = 19.1) for multi-point pseudo-haptic interaction, (61.75 s, SD = 25.1) for single-point pseudo-haptic interaction using a tablet and an S-pen as the input device. The result of Shapiro-Wilk normality test showed that only the elapsed time of vibration feedback had a normal distribution (single-point pseudo-haptic interaction: *W* = 0.965, *p* = 0.644; multi-point pseudo-haptic interaction: *W* = 0.905, *p* = 0.050; vibration feedback: *W* = 0.751, *p* = 1.754×10^−4^). Therefore, instead of using student t-test, Kruskal-Wallis rank sum test was used to compare the elapsed time. The test (*Х*^2^ = 14.206, *p* = 8.226×10^−4^) showed that at least one sample stochastically dominates another sample. Dunn’s test with Bonferroni adjustment was then performed and the test results are summarized in Table 3. The results indicate that the multi-point pseudo-haptic interaction required less time than single-point one. They also reveal that the multi-point pseudo-haptic interaction is more time-efficient than the single-point pseudo-haptic interaction. The nodule detection using an interactive virtual environment with single-point pseudo-haptic feedback required significantly longer time than that with vibration feedback, while the nodule detection time of multi-point pseudo-haptic feedback showed no significant difference from that with vibration-based haptic feedback. When enhanced using multi-point feedback, the interactive virtual environment for soft object stiffness simulation with pseudo-haptic feedback could achieve the same level of accuracy and time efficiency as was the case for vibration-based haptic feedback.

**Table 3:** Dunn’s tests with Bonferroni adjustment for elapsed time of nodule detection using vibration feedback and pseudo-haptic feedback.

Item	*Z*	*p*
*Single-point vs*. *Multi-point pseudo-haptic feedback*	2.451	0.0214*
*Single-point pseudo-haptic feedback vs*. *Vibration*	3.705	0.0003**
*Multi-point pseudo-haptic feedback vs*. *Vibration*	1.255	0.3144

*. Significant at the 5% level;

**. Stronger significance than at the 5% level

### Discussion

We have characterized the user experience with interactive virtual environments via indicators including how well the user performs an interactive task within a particular virtual environment and how long it takes the user to complete the task. The combination of modification of the cursor speed and visualization of the surface deformation resulted in the best performance with regards to (1) nodule detection as shown by the following parameters, true positive rate *Se*, true negative rate *Sp*, probability of true results *ACC* and elapsed time, and (2) in the post-experiment survey. Although the difference among these feedback modalities was not significant regarding true negative rate, the combined feedback showed a significantly better performance on elapsed time and the post-experiment survey than the individual feedback modalities. When employing the cursor speed control only approach, less time was used and a higher true positive rate and probability of true results was achieved when compared to only employing the visualization of the surface deformation approach during the nodule detection experiments. The test results revealed that the visualization of soft surface deformation and the modification of the cursor speed both played an important role in stiffness perception during interactions with the simulated soft object surface. We conclude that it is beneficial to provide both types of feedbacks during the interaction with virtual soft object surfaces.

Single-point and multi-point pseudo-haptic feedback methods showed similar hard nodule detection sensitivities, whilst the multi-point haptic feedback method proved to have a better time efficiency during the nodule detection process. This may be due to a wider exploration area provided by the multi-point feedback method; participants could save time in exploring the surface, comprehending more easily the difference between neighboring surface areas. As observed during the experiments using the single-point pseudo-haptic feedback method, participants sometimes needed to rescan an entire surface area, when they “noticed” the display of an ‘abnormal’ region, to confirm their findings.

The commonly used operation system of tablet computers and smart phones Android allows developers to modulate vibration duration rather than vibration intensity or frequency [37]. With specially designed devices such as stylus with Haptuator [38] and HaCHIStick [39] not only can vibration duration be modulated but also vibration intensity. The purpose of the comparison in this study is to prove our proposed method to be an effective alternative to vibration feedback in current off-the-shelf consumer devices. Therefore, the pseudo-haptic feedback system is benchmarked against vibration feedback provided by an off-the-shelf tablet computer instead of a custom device. Further assessment of the role of vibration intensity (amplitude), frequency and time duration in conveying soft object stiffness information could be considered in future studies. Also, the ability of the vibration feedback method in conveying the hard nodule size information buried under a simulated soft surface should also be investigated further.

## Conclusions

This paper proposes and evaluates a cost-effective method to convey soft object surface stiffness information via combined visualization of soft surface deformation and modification of the indenter avatar speed when passing over an area with higher stiffness. The experimental results indicate that the visualization of the surface deformation of a soft object and the control of the cursor speed both play an important role in stiffness perception during interactions with the simulated soft object surface. In this study, the effectiveness of our pseudo-haptic feedback method, which enables the user to distinguish sizes of virtual hard nodules buried under a simulated object surface using a tablet computer, has been confirmed. To further improve the interactive experience, a multi-point pseudo-haptic feedback method has been conceived and implemented; the experimental results reveal that multi-point pseudo-haptic interaction is more time-efficient than single-point pseudo-haptic interaction. The outcomes of an experimental study comparing vibration-based feedback available in consumer devices with the proposed pseudo-haptic feedback approaches show that multi-point pseudo-haptic feedback performs similar to vibration-based feedback with regards to the elapsed time and the nodule detection sensitivity. The work shows that the proposed pseudo-haptic feedback is a low-cost method to provide an adequate perception of the stiffness of soft objects and hard inclusions embedded within, and that our approach is suitable to be used in many low-budget applications where haptic sensation is required, such as video games and surgical training systems.

## Supporting Information

S1 Table Nodule identification results of three pseudo-haptic techniques. (DOC)

S2 Table Elapsed time of comparison of deformation visualization and cursor speed modification (unit: s). (DOC)

S3 Table Size discrimination result. (DOC)

S4 Table Nodule localization result. (DOC)
