# Supplementary material for: Evaluation of Pseudo-Haptic Interactions with Soft Objects in Virtual Environments
Source: PLoS One. 2016 Jun 28;11(6):e0157681. doi: 10.1371/journal.pone.0157681 (PMC4924842; doi:10.1371/journal.pone.0157681)
Supplement: S1 Table — (DOC) [file pone.0157681.s001.doc]

**S1 Table. Nodule identification results of three pseudo-haptic techniques.**

|  | **True Positive** | | | **False Positive** | | | **False Negative** | | | **True Negative** | | |
| --- | --- | --- | --- | --- | --- | --- | --- | --- | --- | --- | --- | --- |
|  | Deformation | Speed | Combination | Deformation | Speed | Combination | Deformation | Speed | Combination | Deformation | Speed | Combination |
| p1 | 14 | 18 | 17 | 0 | 0 | 0 | 4 | 0 | 1 | 8 | 8 | 8 |
| p2 | 9 | 17 | 16 | 0 | 0 | 0 | 9 | 1 | 2 | 8 | 8 | 8 |
| p3 | 9 | 16 | 17 | 1 | 0 | 0 | 9 | 2 | 1 | 7 | 8 | 8 |
| p4 | 9 | 17 | 17 | 0 | 1 | 0 | 9 | 1 | 1 | 8 | 7 | 8 |
| p5 | 16 | 17 | 17 | 0 | 0 | 0 | 2 | 1 | 1 | 8 | 8 | 8 |
| p6 | 13 | 18 | 17 | 0 | 0 | 0 | 5 | 0 | 1 | 8 | 8 | 8 |
| p7 | 8 | 15 | 16 | 0 | 0 | 0 | 10 | 3 | 2 | 8 | 8 | 8 |
| p8 | 14 | 18 | 17 | 0 | 4 | 0 | 4 | 0 | 1 | 8 | 4 | 8 |
| p9 | 17 | 17 | 18 | 0 | 0 | 0 | 1 | 1 | 0 | 8 | 8 | 8 |
| p10 | 16 | 17 | 17 | 0 | 0 | 0 | 2 | 1 | 1 | 8 | 8 | 8 |
| p11 | 16 | 16 | 18 | 0 | 0 | 0 | 2 | 2 | 0 | 8 | 8 | 8 |
| p12 | 10 | 14 | 17 | 0 | 0 | 0 | 8 | 4 | 1 | 8 | 8 | 8 |
| p13 | 17 | 18 | 18 | 0 | 0 | 0 | 1 | 0 | 0 | 8 | 8 | 8 |
| p14 | 15 | 18 | 17 | 0 | 0 | 0 | 3 | 0 | 1 | 8 | 8 | 8 |
