# Supplementary material for: Evaluation of Pseudo-Haptic Interactions with Soft Objects in Virtual Environments
Source: PLoS One. 2016 Jun 28;11(6):e0157681. doi: 10.1371/journal.pone.0157681 (PMC4924842; doi:10.1371/journal.pone.0157681)
Supplement: S2 Table — (DOC) [file pone.0157681.s002.doc]

**S2 Table. Elapsed time of comparison of deformation visualization and cursor speed modification (unit: s).**

| **Test no.** | **Participant** | **Deformation** | **speed** | **combination** | **Participant** | **deformation** | **speed** | **combination** | **Participant** | **deformation** | **speed** | **combination** |
| --- | --- | --- | --- | --- | --- | --- | --- | --- | --- | --- | --- | --- |
| 1 | p1 | 9 | 9 | 12 | p4 | 17 | 20 | 14 | p7 | 10 | 31 | 50 |
| 2 | p1 | 14 | 9 | 11 | p4 | 24 | 18 | 17 | p7 | 49 | 35 | 38 |
| 3 | p1 | 15 | 9 | 13 | p4 | 19 | 20 | 16 | p7 | 37 | 37 | 40 |
| 4 | p1 | 13 | 11 | 8 | p4 | 20 | 35 | 15 | p7 | 25 | 51 | 26 |
| 5 | p1 | 13 | 8 | 11 | p4 | 12 | 23 | 19 | p7 | 26 | 33 | 28 |
| 6 | p1 | 10 | 10 | 11 | p4 | 20 | 20 | 29 | p7 | 16 | 36 | 30 |
| 7 | p1 | 13 | 9 | 9 | p4 | 16 | 28 | 15 | p7 | 43 | 52 | 29 |
| 8 | p1 | 12 | 8 | 10 | p4 | 15 | 15 | 20 | p7 | 39 | 22 | 10 |
| 9 | p1 | 12 | 11 | 7 | p4 | 24 | 23 | 14 | p7 | 26 | 20 | 9 |
| 10 | p1 | 12 | 10 | 9 | p4 | 22 | 22 | 18 | p7 | 27 | 14 | 24 |
| 11 | p1 | 15 | 9 | 11 | p4 | 50 | 26 | 21 | p7 | 55 | 24 | 28 |
| 12 | p1 | 10 | 10 | 10 | p4 | 20 | 37 | 26 | p7 | 47 | 18 | 21 |
| 13 | p1 | 12 | 9 | 8 | p4 | 21 | 19 | 30 | p7 | 37 | 7 | 7 |
| 1 | p2 | 29 | 25 | 22 | p5 | 7 | 6 | 16 | p8 | 39 | 65 | 40 |
| 2 | p2 | 34 | 19 | 39 | p5 | 20 | 8 | 24 | p8 | 44 | 26 | 20 |
| 3 | p2 | 52 | 37 | 26 | p5 | 31 | 4 | 30 | p8 | 35 | 39 | 26 |
| 4 | p2 | 23 | 42 | 25 | p5 | 13 | 7 | 21 | p8 | 38 | 20 | 20 |
| 5 | p2 | 20 | 30 | 16 | p5 | 13 | 6 | 12 | p8 | 26 | 19 | 16 |
| 6 | p2 | 18 | 20 | 14 | p5 | 7 | 6 | 10 | p8 | 14 | 26 | 25 |
| 7 | p2 | 18 | 25 | 15 | p5 | 28 | 6 | 11 | p8 | 22 | 40 | 22 |
| 8 | p2 | 13 | 30 | 11 | p5 | 14 | 5 | 4 | p8 | 22 | 17 | 11 |
| 9 | p2 | 18 | 14 | 10 | p5 | 10 | 13 | 3 | p8 | 25 | 15 | 11 |
| 10 | p2 | 23 | 10 | 12 | p5 | 5 | 5 | 8 | p8 | 18 | 12 | 11 |
| 11 | p2 | 27 | 22 | 11 | p5 | 13 | 6 | 4 | p8 | 41 | 9 | 9 |
| 12 | p2 | 23 | 19 | 13 | p5 | 31 | 9 | 7 | p8 | 23 | 16 | 27 |
| 13 | p2 | 34 | 16 | 11 | p5 | 18 | 3 | 5 | p8 | 24 | 21 | 16 |
| 1 | p3 | 15 | 24 | 11 | p6 | 11 | 20 | 18 | p9 | 14 | 22 | 49 |
| 2 | p3 | 17 | 17 | 12 | p6 | 22 | 24 | 15 | p9 | 33 | 19 | 18 |
| 3 | p3 | 20 | 20 | 7 | p6 | 16 | 26 | 16 | p9 | 14 | 16 | 18 |
| 4 | p3 | 25 | 16 | 11 | p6 | 19 | 14 | 14 | p9 | 15 | 44 | 10 |
| 5 | p3 | 12 | 13 | 9 | p6 | 17 | 20 | 20 | p9 | 17 | 13 | 16 |
| 6 | p3 | 12 | 12 | 8 | p6 | 16 | 12 | 15 | p9 | 11 | 13 | 12 |
| 7 | p3 | 10 | 13 | 11 | p6 | 16 | 23 | 10 | p9 | 42 | 18 | 18 |
| 8 | p3 | 12 | 9 | 11 | p6 | 22 | 12 | 18 | p9 | 18 | 11 | 12 |
| 9 | p3 | 17 | 12 | 10 | p6 | 20 | 15 | 14 | p9 | 15 | 13 | 10 |
| 10 | p3 | 22 | 11 | 16 | p6 | 18 | 11 | 26 | p9 | 10 | 16 | 8 |
| 11 | p3 | 24 | 11 | 10 | p6 | 12 | 13 | 17 | p9 | 38 | 24 | 10 |
| 12 | p3 | 17 | 13 | 11 | p6 | 15 | 15 | 10 | p9 | 18 | 13 | 9 |
| 13 | p3 | 29 | 11 | 9 | p6 | 22 | 16 | 14 | p9 | 25 | 11 | 16 |
| 1 | p10 | 20 | 12 | 14 | p12 | 9 | 13 | 12 | p14 | 31 | 23 | 23 |
| 2 | p10 | 20 | 11 | 17 | p12 | 11 | 17 | 11 | p14 | 27 | 19 | 24 |
| 3 | p10 | 23 | 14 | 18 | p12 | 11 | 10 | 12 | p14 | 22 | 27 | 22 |
| 4 | p10 | 24 | 15 | 15 | p12 | 11 | 17 | 13 | p14 | 20 | 35 | 20 |
| 5 | p10 | 20 | 14 | 12 | p12 | 17 | 11 | 16 | p14 | 19 | 26 | 22 |
| 6 | p10 | 14 | 11 | 9 | p12 | 10 | 13 | 18 | p14 | 17 | 22 | 25 |
| 7 | p10 | 15 | 13 | 12 | p12 | 14 | 22 | 19 | p14 | 37 | 25 | 26 |
| 8 | p10 | 16 | 9 | 18 | p12 | 12 | 33 | 13 | p14 | 20 | 16 | 16 |
| 9 | p10 | 12 | 6 | 10 | p12 | 15 | 15 | 9 | p14 | 19 | 15 | 16 |
| 10 | p10 | 9 | 8 | 8 | p12 | 11 | 9 | 25 | p14 | 13 | 13 | 17 |
| 11 | p10 | 15 | 9 | 7 | p12 | 20 | 13 | 16 | p14 | 30 | 15 | 22 |
| 12 | p10 | 14 | 7 | 5 | p12 | 13 | 17 | 9 | p14 | 16 | 24 | 17 |
| 13 | p10 | 13 | 6 | 12 | p12 | 13 | 11 | 20 | p14 | 27 | 12 | 25 |
| 1 | p11 | 18 | 12 | 14 | p13 | 16 | 24 | 17 |  |  |  |  |
| 2 | p11 | 12 | 25 | 13 | p13 | 21 | 18 | 19 |  |  |  |  |
| 3 | p11 | 25 | 13 | 20 | p13 | 26 | 22 | 17 |  |  |  |  |
| 4 | p11 | 18 | 15 | 11 | p13 | 24 | 18 | 15 |  |  |  |  |
| 5 | p11 | 9 | 17 | 12 | p13 | 16 | 13 | 15 |  |  |  |  |
| 6 | p11 | 16 | 10 | 20 | p13 | 14 | 18 | 13 |  |  |  |  |
| 7 | p11 | 14 | 11 | 17 | p13 | 15 | 14 | 10 |  |  |  |  |
| 8 | p11 | 18 | 18 | 12 | p13 | 14 | 12 | 10 |  |  |  |  |
| 9 | p11 | 29 | 8 | 12 | p13 | 13 | 12 | 9 |  |  |  |  |
| 10 | p11 | 11 | 5 | 9 | p13 | 11 | 9 | 12 |  |  |  |  |
| 11 | p11 | 30 | 9 | 9 | p13 | 16 | 16 | 8 |  |  |  |  |
| 12 | p11 | 27 | 8 | 10 | p13 | 9 | 10 | 12 |  |  |  |  |
| 13 | p11 | 18 | 5 | 16 | p13 | 8 | 12 | 11 |  |  |  |  |
