# Supplementary material for: Evaluation of Pseudo-Haptic Interactions with Soft Objects in Virtual Environments
Source: PLoS One. 2016 Jun 28;11(6):e0157681. doi: 10.1371/journal.pone.0157681 (PMC4924842; doi:10.1371/journal.pone.0157681)
Supplement: S3 Table — (DOC) [file pone.0157681.s003.doc]

**S3 Table. Size discrimination result.**

|  | **Recognized order (A>B>C, 1: largest, 3: smallest)** | | |
| --- | --- | --- | --- |
| **Participant** | **A** | **B** | **C** |
| p1 | 1 | 3 | 2 |
| p2 | 1 | 3 | 2 |
| p3 | 2 | 3 | 1 |
| p4 | 2 | 1 | 3 |
| p5 | 1 | 2 | 3 |
| p6 | 1 | 2 | 3 |
| p7 | 1 | 2 | 3 |
| p8 | 1 | 3 | 2 |
| p9 | 3 | 2 | 1 |
| p10 | 1 | 2 | 3 |
| p11 | 1 | 2 | 3 |
| p12 | 1 | 3 | 2 |
| p13 | 1 | 2 | 3 |
| p14 | 1 | 2 | 3 |
| p15 | 1 | 2 | 3 |
| p16 | 1 | 2 | 3 |
| p17 | 1 | 2 | 3 |
| p18 | 1 | 2 | 3 |
| p19 | 2 | 1 | 3 |
| p20 | 1 | 2 | 3 |
