# Supplementary material for: Evaluation of Pseudo-Haptic Interactions with Soft Objects in Virtual Environments
Source: PLoS One. 2016 Jun 28;11(6):e0157681. doi: 10.1371/journal.pone.0157681 (PMC4924842; doi:10.1371/journal.pone.0157681)
Supplement: S4 Table — (DOC) [file pone.0157681.s004.doc]

S4 Table. Nodule localization result.

|  | **Single-point pseudo-haptic feedback** | | | | **Multi-point pseudo-haptic feedback** | | | | **Vibration feedback** | | | |
| --- | --- | --- | --- | --- | --- | --- | --- | --- | --- | --- | --- | --- |
| **Participant** | **A** | **B** | **C** | **Time (s)** | **A** | **B** | **C** | **Time (s)** | **A** | **B** | **C** | **Time (s)** |
| p1 | 1 | 1 | 1 | 97 | 1 | 1 | 1 | 67 | 1 | 1 | 1 | 22 |
| p2 | 1 | 1 | 0 | 51 | 1 | 1 | 0 | 18 | 1 | 1 | 1 | 34 |
| p3 | 1 | 1 | 1 | 113 | 1 | 1 | 0 | 73 | 1 | 1 | 1 | 101 |
| p4 | 1 | 1 | 0 | 74 | 1 | 1 | 0 | 50 | 1 | 1 | 0 | 15 |
| p5 | 1 | 1 | 0 | 23 | 1 | 1 | 1 | 23 | 1 | 1 | 1 | 24 |
| p6 | 1 | 1 | 1 | 90 | 1 | 1 | 1 | 68 | 1 | 1 | 1 | 28 |
| p7 | 1 | 1 | 1 | 40 | 0 | 1 | 1 | 27 | 1 | 1 | 1 | 24 |
| p8 | 1 | 1 | 1 | 91 | 1 | 1 | 1 | 50 | 1 | 1 | 1 | 16 |
| p9 | 1 | 1 | 1 | 73 | 1 | 1 | 1 | 18 | 1 | 1 | 1 | 56 |
| p10 | 1 | 1 | 0 | 62 | 1 | 1 | 1 | 37 | 1 | 1 | 1 | 44 |
| p11 | 1 | 1 | 1 | 29 | 1 | 1 | 1 | 21 | 1 | 1 | 1 | 43 |
| p12 | 1 | 1 | 1 | 56 | 1 | 1 | 1 | 45 | 1 | 1 | 1 | 34 |
| p13 | 1 | 1 | 1 | 67 | 1 | 0 | 1 | 67 | 1 | 1 | 0 | 45 |
| p14 | 1 | 1 | 0 | 44 | 1 | 1 | 1 | 54 | 1 | 1 | 0 | 34 |
| p15 | 1 | 1 | 1 | 32 | 1 | 1 | 1 | 28 | 1 | 1 | 1 | 26 |
| p16 | 1 | 1 | 1 | 66 | 1 | 1 | 1 | 40 | 1 | 1 | 1 | 27 |
| p17 | 1 | 1 | 1 | 40 | 1 | 1 | 1 | 20 | 1 | 1 | 1 | 25 |
| p18 | 1 | 1 | 1 | 89 | 1 | 1 | 0 | 64 | 1 | 1 | 1 | 17 |
| p19 | 1 | 1 | 1 | 58 | 1 | 1 | 0 | 40 | 1 | 1 | 1 | 30 |
| p20 | 1 | 1 | 1 | 40 | 1 | 1 | 1 | 23 | 1 | 1 | 1 | 34 |
